# A Conceptual Framework for Evaluating National Organ Donation and Transplantation Programs

**DOI:** 10.3389/ti.2023.11006

**Published:** 2023-05-25

**Authors:** Charlotte Johnston-Webber, Jasmine Mah, Simon Streit, Apostolos Prionas, George Wharton, Elias Mossialos, Vassilios Papalois

**Affiliations:** ^1^ Department of Health Policy, London School of Economics and Political Science, London, United Kingdom; ^2^ Department of Medicine, Dalhousie University, Halifax, NS, Canada; ^3^ Department of Surgery, Imperial College, London, United Kingdom; ^4^ Department of General Surgery, Whipps Cross Hospital, Barts Health NHS Trust, London, United Kingdom; ^5^ Institute of Global Health Innovation, Imperial College, London, United Kingdom; ^6^ Renal and Transplant Unit, Hammersmith Hospital, Imperial College Healthcare NHS Trust, London, United Kingdom

**Keywords:** organ donation, organ transplant, comparative analysis, transplantation policy, transplant system

## Abstract

Conceptual frameworks are valuable resources that can be used to guide the planning, evaluation, and development of healthcare services. However, there are currently no comprehensive frameworks focused on organ donation and transplantation that identify the critical factors underlying a successful national program. To address this knowledge gap, we developed a conceptual framework that takes into account all major domains of influence, including political and societal aspects as well as clinical implementation. The framework was initially constructed based on a targeted review of the relevant medical literature. Feedback provided by a panel of international experts was incorporated into the framework *via* an iterative process. The final framework features 16 essential domains that are critical for initiating and maintaining a successful program and improving the health of patients with organ failure. Of particular note, these domains are subject to three overarching health system principles: responsiveness, efficiency, and equity. This framework represents a first attempt to develop a whole-system view of the various factors that contribute to the success of a national program. These findings provide a useful tool that can be adapted to any jurisdiction and used to plan, evaluate, and improve organ donation and transplantation programs.

## Introduction

Conceptual frameworks have important roles in guiding the development of complex processes. These frameworks may be particularly helpful in healthcare systems because they can present a method that can be used to consider the many different factors that influence policy development, implementation, and outcomes. There is a growing interest in the use of frameworks to evaluate healthcare system performance and quality (1) and to assist in their planning and development (2). Solid organ donation and transplantation is a good example of a complex healthcare process, as it involves multiple, frequently time-limited steps and requires high-level coordination and collaboration between distinct stakeholders. Moreover, trust and confidence from the general public must be established and maintained in order to achieve success. The challenges involved in this process extend far beyond those associated with basic clinical practice or a single healthcare system alone. Many interrelated factors must be addressed to develop and sustain a high-quality program that meets the needs of the population.

Given these inherent complexities, the successful development of an equitable and efficient national organ donation and transplantation program remains challenging for many jurisdictions. A focused conceptual framework will be an essential tool that will help policymakers and healthcare leaders to implement the numerous and intricate features of this discipline. A conceptual framework can be used as a means to evaluate a program, identify gaps in services provided, and formulate targeted plans for reform and development. It can also be used to guide the establishment of structures and processes that facilitate continuous quality improvement and effective and sustainable investment.

The current medical literature includes many academic papers and clinical guidelines that address one or more aspects of organ donation and transplantation. However, to the best of our knowledge, none of these publications provide a framework with a comprehensive system-wide view of the critical components of an organ donation and transplantation program. Thus, the objective of this paper is to provide such a framework. Specifically, our framework was designed to be adaptable to any setting and contains all the components necessary to establish a successful solid organ donation and transplantation program.

## Materials and Methods

The framework was developed *via* an iterative process in three main stages. In stage one, we performed a targeted narrative review of the relevant literature. We identified several key documents and resources recognized as important internationally in the field of organ donation and transplantation (Table 1). These sources helped us to identify the main components or domains of a successful organ donation and transplantation system. The references cited in these publications were also reviewed for more detailed information. Appropriate keywords and phrases relevant to the main domains, such as “organ donation,” “transplantation,” “deceased donation,” “live donation,” “post-transplant follow-up” and “national transplant organization” were generated from this full set of publications and used to identify additional sources of information to be included in our review. Additional relevant publications were retrieved from databases including Medline and Web of Science. Internet search engines (e.g., Google Scholar) were also used to retrieve relevant papers from the academic literature. While the searches were not limited by year of publication, non-English language publications were excluded from further consideration. One researcher screened the titles and abstracts and final selections were made based on relevance to the identified domains. Additional hand-searches of references cited by the included studies were also undertaken. These searches also facilitated the retrieval of relevant items from the grey literature, including international reports and reviews. Our searches of the grey literature were not limited by year of publication but were limited to English language publications. This information was used to refine and develop the domains of the framework as well as to identify their interrelationships.

**TABLE 1:** Examples of key documents and resources of international importance identified as part of the targeted literature review.

Title/Organization	References
European Directorate for the Quality of Medicines (EDQM):	
Guide to Quality and Safety of Organs for Transplantation; 7th edition	(3)
Transplant newsletters	(4)
ODEQUS^a^ Quality Criteria and Quality Indicators in Organ Donation	(5)
Eurotransplant	(6)
KDIGO	(7–9)
American Transplantation Society	
British Transplantation Society	(10–13)
European Society for Organ Transplantation	
UEMS^c^ – Division of Transplantation	
NHS Blood and Transplant resources	
ERA-EDTA^d^ registry reports	(14)
World Health Organization	(15)
Madrid Resolution on Organ Donation and Transplantation	(16)
The Declaration of Istanbul on Organ Trafficking and Transplant Tourism	(17)

^a^ Organ Donation European Quality System.

^b^ Kidney Disease Improving Global Outcomes.

^c^ Union Européenne des Médecins Spécialistes.

^d^ European Renal Association - European Dialysis and Transplant Association.

As part of a wider project and parallel to the development of the framework, the research team also created profiles of programs currently in use in six European countries, including Croatia, Greece, Italy, Portugal, Spain, and the United Kingdom (18–23). The findings from these parallel research studies also provided insight into the development of this conceptual framework.

The second stage of the iterative process included an open collaborative discussion between the authors. This process ultimately led to the construction of the first draft of the conceptual framework. A consensus was reached on the 16 key domains to be included.

The third stage included consultation with a panel of international experts in organ donation and transplantation (Appendix 1). Due to the restrictions placed on our activities during the COVID-19 pandemic, these discussions were conducted in a series of virtual meetings with single individuals and with all experts. Over the course of two rounds of feedback, the expert panel provided suggestions for additions and modifications to our framework. Their feedback was incorporated *via* an iterative process which led to the final version of the framework.

## Results

The first iteration of the framework included 12 domains that were identified based on the results of the literature review and open discussion among the seven authors. Each domain represents an essential component of a successful organ donation and transplantation program. Following the first round of expert feedback and further collaborative discussion, five additional domains were added to this list (Table 2). The prevailing opinion was that several of the elements of the original 12 domains were sufficiently important to be included as unique entities.

**TABLE 2:** Domains included after the first and second framework iterations. Additions included after the second iteration are shown in bold.

Government: political support and long-term commitment
Key legislation
Reducing the need for transplant: a whole-system approach
Building and maintaining public support and trust in the system
The National Transplant Organization
Reimbursement mechanisms for staff and facilities
Infrastructure
Deceased donation
Living donation
Transplantation
Post-transplant follow-up
Patient-centered care
Quality standards and quality improvement
Databases and information technology
Teaching, training, and professional development
Research and development
Professional organizations and scientific societies

**FIGURE 1:**
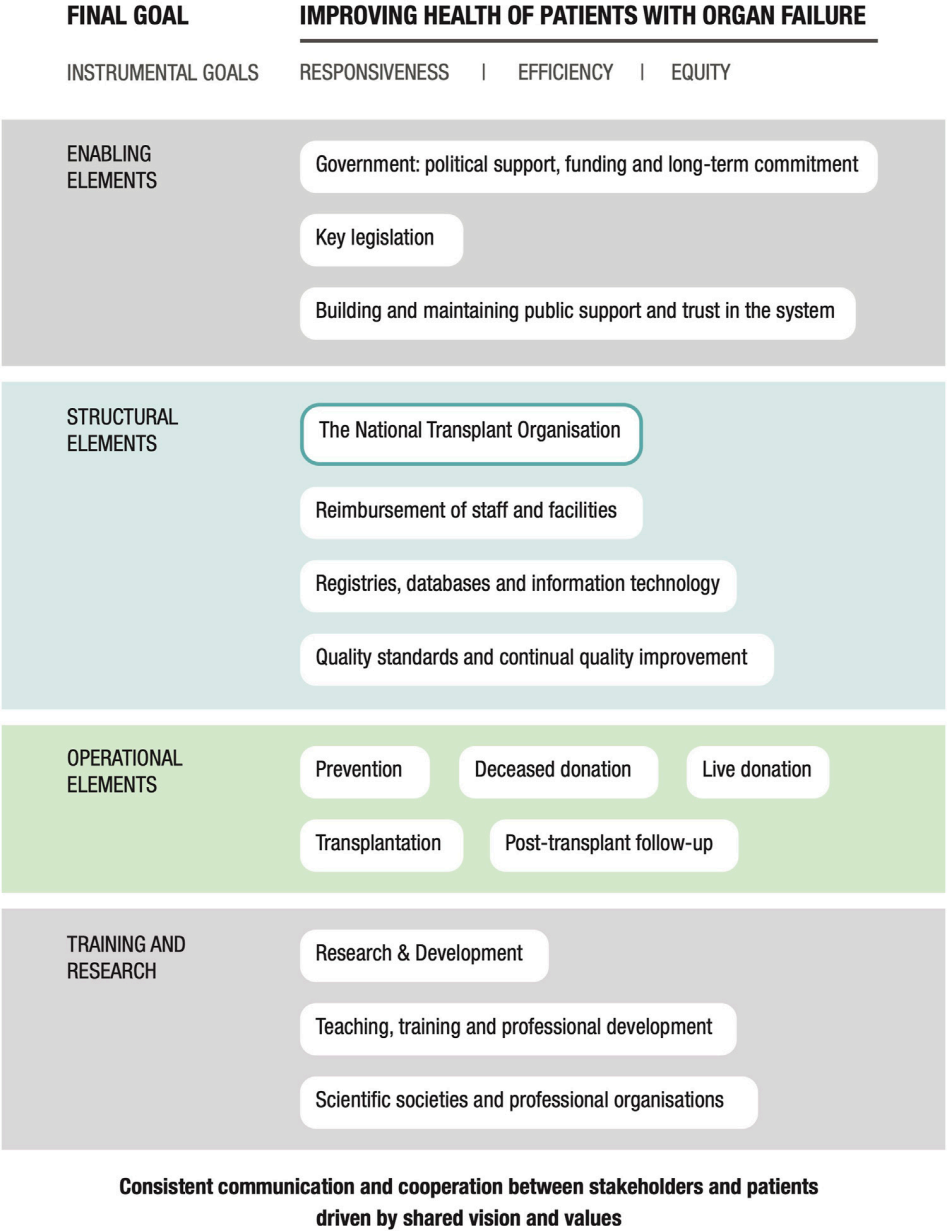
A conceptual framework for a national solid organ donation and transplantation program.

Following further discussion and expert review, a third and final iteration of the framework was performed. This involved the organization of the domains into a diagram that revealed their relationships with one another (Figure 1). As shown, all researchers and expert participants agreed that the overall goal of any program should be the “Improved health of patients with organ failure.” The instrumental goals listed below this, those of Responsiveness, Equity, and Efficiency of donation and transplantation services, are goals in and of themselves, and also means by which this final goal is achieved. This methodology is based on the approach proposed by the World Health Organization (WHO) in their report entitled “Strengthening Health Systems to Improve Health Outcomes” (24) and from the concept groupings identified by Klassen et al. (25) in their systematic review of performance measurements and improvement frameworks in health, education, and social services systems. This approach aims to illustrate how the different components of the framework function as part of a dynamic system as well as their impact on both qualitative and quantitative outcomes. Each domain included in the diagram in Figure 1 makes an important contribution to a successful organ donation and transplantation program.

The 16 domains essential to achieving this overall goal are included in the center of the diagram. The domain count was reduced from 17 to 16 in the final iteration as the domain entitled “Patient-centered care” was subsumed into the instrumental goal of “Responsiveness.” Finally, all elements of the framework are underpinned by consistent communication and collaboration between all stakeholders and patients.

### Final Goal

#### Improving Health of Patients With Organ Failure

Improving the health of patients with organ failure should be the main goal of any organ donation and transplantation program. This includes the implementation of robust programs aimed at reducing the incidence of organ failure and thus the need for transplant (Prevention), as well as efforts to increase rates of organ donation and transplantation and optimize follow-up care. It is important to recognize that the strategies used to increase rates of organ donation and transplantation should not compromise quality and must be coupled with efforts to ensure ongoing improvement and patient satisfaction.

### Instrumental Goals

#### Responsiveness

The World Health Report 2000 identified “Responsiveness” as one of the three critical goals of a healthcare system (26). Responsiveness, as defined by the WHO is:

How well the health system meets the legitimate expectations of the population for the non-health enhancing aspects of the health system. It includes seven elements: dignity, confidentiality, autonomy, prompt attention, social support, basic amenities, and choice of provider (27).

##### Responsiveness to the General Population, Patients, Caregivers, and Their Loved Ones

Creating a program that is both responsive and patient-centered will be vital to any efforts focused on engendering public support and trust. The needs of patients and their caregivers or representatives need to be at the center of any organ donation and transplantation program and these individuals must be recognized as the most important stakeholders on both sides of this process. Patients and their caregivers or representatives should be involved at every level and their input in planning and developing healthcare interventions must be recognized as invaluable to this process. Patient-centered care has a positive impact on the rates of both living and deceased donations (28,29). Patient-centered care may also help to provide potential recipients with an understanding of the risks and benefits of transplantation and may improve medication compliance and self-management (30). Several core concepts of patient-centered care are listed in Table 3.

**TABLE 3:** Core concepts of patient-centered care (31–35).

• Providing collaborative, personalized, and well-coordinated care
• Treating individuals with dignity, compassion, and respect
• Respecting individual choices, preferences, values, culture, and religious beliefs
• Including family and loved ones in the decision-making process
• Taking into account emotional, social, and practical issues
• Making decisions with patients, not for them, and reaching mutual agreement on goals and expectations
• Ensuring prompt, full, and transparent sharing of information
• Improving health literacy to facilitate shared decision-making

Many different strategies can be used to achieve patient-centered care including, (a) providing tailor-made, holistic support including shared electronic health records (36), and telemedicine technology for patients in remote locations (37); (b) conducting regular surveys of the patient and caregiver experiences (32, 33); (c) involving patients and caregivers at all levels of the system (38); and (d) ensuring patient and caregiver input to the planning and development of services and educational curricula for healthcare staff (39).

##### Responsiveness to staff

Recruitment and retention of a well-supported and motivated workforce are crucial aspects of efforts to build a functional and responsive healthcare system (40). The needs of staff must be addressed by optimizing the work environment and providing high-quality training and development opportunities. Workload expectations must be reasonable with adequate staffing to cover the full patient roster as well as time set aside for training and other continued professional development activities. It will also be crucial to provide adequate reimbursement for specific duties and to recognize outstanding contributions and achievements. There should be clear leadership hierarchies and trainees must have access to appropriate supervision and support with structures in place designed to nurture a collegial culture of shared learning and development.

#### Efficiency and Equity

As in all other areas of healthcare, a national donation and transplantation program must strive to use all resources at its disposal as efficiently as possible and to the maximum benefit of its target patient population (41). Of equal importance, but perhaps posing a greater challenge, a successful program must consider the principle of equity in all organ donation and transplantation activities. The supply of organs available for transplant is outstripped by need; thus, there must be measures in place that ensure that this scarce resource is distributed based on clinical need and in accordance with justified and respected national criteria (9, 18, 37). Access to transplantation must be equitable and not influenced by the ability to pay, personal connections, or other privileged circumstances. There should also be equity in organ donation; the state can play an important role in ensuring that all segments of the population are well-informed about organ donation and that systematic disparities are minimized (3). For example, there are persistent imbalances in the number of organ donors and the number of patients in need of a transplant among ethnic minorities in both the UK and the United States that remain to be addressed (42, 43).

Equity in organ allocation is of paramount importance and involves complex decision-making processes taking many different factors into account. Due to the shortage of viable organs, this means that some difficult decisions often need to be made and a national waiting list is necessary. The guiding principles of the WHO state that rules for allocation must be “equitable, externally justified and transparent” (15). The task of deciding on national allocation rules should be delegated to committees comprising medical, public health, ethics experts and patient representatives and the national organ allocation system must be objective, transparent and fair. It should be subject to oversight by the National Transplant Organisation (NTO), and there must be processes in place to ensure regular audits of the system and adherence to the rules. It is essential that cultural and community values are taken into account and that there should be no discrimination on the basis of a recipient’s race, religion or gender. Tissue type and antibody matching are clearly crucial elements, but other factors such as equity, utility, benefit and fairness are usually taken into account. It Is important that, as far as is possible when taking into account the technical and practical constraints with respect to organ retrieval, preservation and transportation, a patient-based allocation, not a centre-based allocation system is adopted. Different rules will also be required for different types of transplant. For example, in some instances of liver disease patients may die within a few days of organ failure, with transplant being the only effective treatment. Conversely, most cases of kidney failure can be maintained for a period of time on dialysis. Some jurisdictions such as Israel and the USA give priority to those who have been previous living donors, or who have previously given permission for the organs of their loved ones to be donated. However, this kind of priority listing can raise some difficult ethical dilemmas.

### Enabling Elements

#### Government: Political Support, Funding, and Long-Term Commitment

Long-term governmental backing and commitment are essential for the development and maintenance of a successful organ donation and transplantation program. The complex nature of organ donation and transplantation will require high-level collaboration across the healthcare system as well as attention to smaller details at an operational level. All stakeholders must be united in a shared vision of how to achieve the desired goals; this will require clear, decisive, and consistent leadership.

Governmental support must be accompanied by adequate and sustainable sources of funding. Ultimately, an effective donation and transplantation program will deliver profound improvements to the patients’ quality of life as well as savings to the healthcare system and financial benefits to the public at large. However, building a comprehensive program requires considerable financial investment to ensure that all the necessary components are suited for their intended purpose and that adequate staffing and resources are provided.

#### Key Legislation

Legislation should address all the possible modes of organ donation as well as the determination of death by neurological criteria and the national consent policy (9, 18, 38). Protection against organ trafficking or coercion must be in place for those lacking capacity to consent (17). It is also desirable to have key staff roles and responsibilities outlined clearly together with a clear indication of the minimum staffing levels required by participating units based on their size and anticipated level of activity. Legislation should guide and facilitate this process and not present an undue impediment to organ donation or transplantation (44). Changes should be made only after consultation with all stakeholders including the general public (45) to prevent the occurrence of unintended consequences (Example 1).

Example 1 Two contrasting examples of changes to consent legislation: Wales and Greece.

“Opt-out” consent policies have become popular in recent years. Greece enacted this type of legislation in 2013. Wales followed with similar legislation in 2015, albeit with contrasting outcomes. Before the enactment of this legislation, all stakeholders in Wales participated in a comprehensive consultation period that included a survey of opinions from the general public (46). This was followed by a multimodal publicity campaign designed to ensure that the public understood the changes in the law, which included the stipulation that a patient’s relatives would continue to have the final say in all decisions. While the impact of this legislation was not immediate, recent findings revealed that consent rates in Wales increased steadily in the years following this legislative change. When compared with consent rates reported in England (which did not enact this type of legislation until 2020), the upward trend in consent rates achieved statistical significance after 33 months (47).

By contrast, in Greece, there was minimal consultation with the public and little publicity regarding the practical impact of the changes in these laws on the general population. The unintended consequence of this approach was a backlash against the new legislation, with many Greek citizens actively registering an objection to organ donation on the donor register (48). Largely as a result of this response, in 2018, Greece returned to the earlier “opt-in” consent system. Greece continues to struggle with low rates of organ donation and family consent.

#### Building and Maintaining Public Support and Trust in the System

It is imperative to generate strong public support and trust in a national organ donation and transplantation program. This support can increase over time by strict observance of the highest ethical standards and by adherence to fair, equitable, and transparent processes. Good governance of all aspects of the program is essential. This information can be conveyed *via* regular, publicly available reports of inspections, progress, and activity. Transparency and accountability regarding any untoward events or incidents are also needed to sustain public trust.

Results from previous studies suggest that educational campaigns aimed at the general public have a modest impact in increasing the overall donation rate as well as the number of people who state their willingness to donate their organs (5, 44). These programs should be implemented by the National Transplant Organization (NTO) and might aim to dispel misconceptions and promote principles of social solidarity and altruism. The Madrid Resolution (16) highlighted the need for normative change to support the principles of organ donation and proposed that public education should begin in schools. Evidence suggests that these efforts can change attitudes and boost the number of individuals willing to donate their organs (49). Most religions are supportive of organ donation and transplantation (50–53). However, religious leaders should be consulted before making any changes because some issues, for example, the diagnosis of brain death, remain controversial. Periodic surveys may also help to inform future campaigns and identify issues that need to be addressed.

The press and the media have a powerful influence in shaping public opinion. Media coverage can be utilized to endorse the program by promoting poignant personal stories and highlighting the lives saved by organ transplantation. Positive media coverage is equally or more effective at increasing donation rates than public campaigns aimed at improving knowledge and awareness (54). Example 2 highlights the impact of a media campaign that contributed to a change in consent legislation in England.

Example 2 The powerful influence of media portrayals of organ donation and transplantation: Max and Keira’s law.

In 2017, a young girl in the UK named Keira Ball was killed in a car accident. Her donated heart saved the life of Max Johnson, then 9 years of age, who had been diagnosed with dilated cardiomyopathy. This story was featured prominently in the national tabloid press, most notably in the daily news publication, *The Mirror*. With this story, *The Mirror* ran a successful campaign that supported a change to the consent legislation in England from an “opt-in” to an “opt-out” policy. The “opt-out” legislation enacted in 2020 has become known as “Max and Keira’s law.”

### Structural Elements

#### The National Transplant Organization (NTO)

The NTO plays a crucial role in promoting successful organ donation and transplantation programs. International guidance advises that the NTO must be adequately funded and resourced as a single and independent public body (18, 45). The EDQM Guide to the Quality and Safety of Organs for Transplantation (7th edition) provides a comprehensive list of the essential and supplementary functions of an NTO (3). Various countries have adopted different models of operation according to their national priorities (18–22). Two contrasting models are outlined in Example 3.

Example 3 The NHS Blood and Transplant (UK) and the Croatian National Transplant Organization.

The UK has substantially improved its organ donation and transplantation programs *via* a series of successful reforms and initiatives. All activities are now directed by a single, independent Special Health Authority known as the NHS Blood and Transplant (NHSBT). This authority covers all four jurisdictions of the UK and a population of approximately 66.6 million individuals. The NHSBT oversees 12 regional organ donation teams that serve their designated populations and cover several NHS Trusts and/or Boards. The NHSBT is supported by eight solid organ advisory groups, in which representatives of all relevant organizations collectively review activity and outcomes, discuss policy, and set research priorities (55).

By contrast, Croatia is a country with a small population (∼4 million) with a substantially lower healthcare budget. Unlike the other European programs, the Croatian National Transplant Organization (NTO) is directly governed and overseen by the Croatian Ministry of Health and is under the leadership of a single National Transplant Coordinator who was appointed in 2001. The National Transplant Coordinator is supported by a robust team that is responsible for administering a nationwide transplant network. This model is well-suited to this setting and has enabled Croatia to become a world leader in organ donation and transplantation.

#### Reimbursement of Staff and Facilities

Although transplantation is highly cost-effective over the long term (56), it is expensive and resource-intensive to perform. To generate a successful program, no stage of the process should pose a financial burden to any organization and every step must be adequately reimbursed at nationally agreed-upon rates. To ensure equity of access reimbursement schedules must be carefully devised to account for all eventualities including items such as advanced techniques for organ preservation. While financial or other incentives may be useful, they will need to be carefully designed and consistently implemented. Additionally, the work associated with both donation and transplantation often involves long, intensive shifts involving both night and weekend hours. The remuneration provided must also reflect these factors. Example 4 describes two successful reimbursement mechanisms that have been implemented in Croatia and Spain.

Example 4 Reimbursement mechanisms: Croatia and Spain.

The adoption of a new reimbursement scheme (in 2006) is considered to be a key factor in Croatia’s recent success in organ donation and transplantation (22). The costs of organ donation in Croatia are now reimbursed by a special state budget and transplant activities are covered *via* a set of Diagnosis Related Groups (57). This has helped to mitigate the impact of limited financial and other resources and has removed any financial burden that might impede participation in this program. This reimbursement strategy has proven to be especially important for facilitating organ donation in smaller hospitals that would be otherwise unable to sustain a transplant program.

In Spain, some hospitals provide incentive bonuses to medical professionals who facilitate organ donations. This may contribute to Spain’s high rate of organ donation (58). However, caution must be used when considering this strategy. Conflicts of interest may arise if the clinicians engaged in initiating family discussions and identifying potential donors are also involved in their direct clinical care. Wherever possible, the two roles should be separate. It will be imperative to maintain full transparency regarding roles and responsibilities in order to maintain trust in the system.

#### Infrastructure

Infrastructure requirements must be determined based on current and projected future demand. The distribution of the various organizations needs to be considered, particularly given the time-limited nature of many of the processes and the travel times that may be required. All facilities and equipment must be regularly maintained, updated, and replaced when they become obsolete.

#### Registries, Databases, and Information Technology

The national program must be supported by a sophisticated information technology system that is easily accessed by all staff members. The technology system should be overseen by the NTO under the auspices of a chief digital and information technology (IT) officer. The system should aim to integrate all the data necessary for transplant assessments, including organ matching, allocation, transplantation, and long-term follow-up. In many jurisdictions advanced artificial intelligence algorithms are used to improve the efficiency and accuracy of these processes (59, 60). The data collected should meet the requirements of international organ exchange schemes, for example, the Eurotransplant Network Information System of Eurotransplant (61).

#### Quality Standards and Continual Quality Improvement

A quality and safety framework that covers all aspects of donation and transplantation will be needed to ensure that a national program maintains the highest standards and strives for ongoing quality improvement. This is a requirement of the European Union (EU) directive 2010/53/EU (62). Similarly, the Organ Donation European Quality System (ODEQUS) manual on quality criteria and quality indicators (5) provides detailed recommendations across different modes of donation. The EDQM Guide to the Quality and Safety of Organs for Transplantation also provides recommendations on quality criteria and indicators for transplantation programs (3). Formal arrangements for authorization, licensing, and regular inspection of all facilities, appropriate equipment and personnel, and nationally agreed-upon procedures must be established that facilitate improvement in cases in which a given party fails to meet expected standards. The quality and safety framework should be devised, updated, and overseen by the NTO with the advice and support of appropriate experts.

### Operational Elements

#### Prevention

Future needs for transplants and strategies that aim to reduce the incidence of organ failure remain a critical priority (16)*.* Diabetic nephropathy accounts for around 20%–30% of the cases accepted for all forms of renal replacement therapy in most European countries; hypertensive nephrosclerosis accounts for another ∼10%–20% (63). Cirrhosis and primary liver cancer are the leading causes of liver failure, while alcohol consumption, viral hepatitis B and C, and obesity are also among the main causes of these conditions (64).

Prevention strategies must include both primary and secondary measures. Once tertiary preventative measures are needed, patients have already progressed to terminal organ failure. Efforts to establish effective primary and secondary preventative strategies extend beyond the scope of organ donation and transplantation alone and require a robust public health program that is supported by primary healthcare systems that are fully integrated with both secondary and tertiary care. Careful consideration will be needed to determine how a specific donation and transplantation program fits within the wider healthcare system of a given nation.

Thus, efforts to optimize the health of patients nearing end-stage renal failure and those maintained on dialysis will be essential to the success of the program. It is essential that the responsible physicians are regarded as an integral part of the program and are confident in the assessment and referral process for transplant. As outlined above, pre-emptive transplant, prior to the need for dialysis, and ideally from a living donor, should always be the treatment of choice. Pre-emptive transplant, thus avoiding the risks of dialysis and ensuring the patient is in better health at the point of transplant consistently shows better outcomes (65, 66). The possibility of pre-emptive transplant should be an integral part of the discussions around treatment options prior to the development of end-stage renal failure. Additionally, dialysis centers are operated by private providers in many countries; therefore, measures must be in place to ensure that these providers adhere to the highest standards of quality and safety. Example 5 describes how Portugal has successfully restructured dialysis care.

Example 5 Improving the quality of dialysis care in Portugal.

Largely due to specific demographic and epidemiological issues, Portugal has a high incidence and prevalence of patients with end-stage renal disease. Hemodialysis in Portugal is offered primarily by private providers, and, until 2008, it was reimbursed on a fee-for-service basis. This has since been replaced by a capitation prospective payment system that includes information and monitoring systems that can be used to evaluate process and outcome measures. Providers now receive comprehensive bundled per-patient payments which are based on the submission of a set of quality indicators. A National Dialysis Monitoring Commission was established to assess performance and trends. The Portuguese Ministry of Health has reported that these strategies have been successful both in improving the quality of care and containing costs (67, 68).

#### Deceased Donation (DD)

Countries with the highest transplantation rates typically have well-developed DD schemes (3). Donation after Brain Death (DBD) and Donation after Circulatory Death (DCD) both present specific ethical challenges which must be addressed by clear national legislation and guidance (as discussed further below). Although DBD remains the main source of organs available by DD in most countries, DCD now represents a significant source in many countries with high donation rates, notably Spain (69) and the UK (70).

Efforts to maximize the number of donors require ongoing identification and referral. Organ Donation Coordinators (ODCs) play an important role in this process. Previous evidence suggests that ODC involvement is one of the most important elements contributing to family consent to donation (71, 72). ODCs must be specifically trained with protected time for their duties and must be available at all times and every day during the year. General staff training is also important in increasing the rate of DD (73, 74). This is particularly important for staff members assigned to areas where there may be a large number of potential donors, for example, the Intensive Care Unit (ICU). Appropriately-trained clinicians must be available to facilitate DD *via* prompt diagnosis of death and communication of this information to families and loved ones. Information concerning the diagnosis of death or withdrawal of life-saving treatment should not be linked to conversations about organ donation unless the topic is raised spontaneously. Among the strategies that might be used to increase the donor pool, the use of expanded criteria donors and/or non-standard risk donors might be considered. For example, Eurotransplant’s Senior Program matches older donors to older recipients. Spain has successfully employed both strategies (Example 6).

Example 6 Strategies for expanding the donor pool: Spain.

Fortunately, Spain has experienced many fewer traffic accidents in recent years. However, this would have led to fewer organ donations had Spain not initiated the use of expanded criteria and non-standard risk donors as part of the “40 Donors pmp Plan” in 2008. This action has successfully increased the donor pool. In 2015, more than 50% of DDs in Spain were from individuals over 60 years of age. The program preferentially matches older DDs to older recipients. In addition, because of concerns that a significant number of potential donors and organs were being rejected for poorly-substantiated medical reasons, the Spanish NTO established a medical team that is available at all times to provide advice to ODCs that may be unsure of the suitability of a potential donor (75).

Several internationally-recognized documents provide guidelines on the management of DDs from patients in ICUs (76, 77). Donor evaluation and management must be performed using protocols that have been approved at the national level. Donor management must be initiated as soon as possible (78) with evaluation undertaken or supervised by trained specialists following internationally-approved criteria (3). The NTO must be available at all times to provide advice and support for any difficult decisions. Organ retrieval, packing, preservation, and storage should also be performed using nationally-approved protocols. Dedicated teams must be available to perform these functions under the supervision of an NTO. Transportation agreements should also be in place, including those that are needed to facilitate air transport as necessary.

The merits of different techniques of organ optimisation and preservation are still to be fully determined, and trials and evaluations comparing various approaches are ongoing (3). However, successful methods promise to deliver improvements in graft survival time, immediate and long-term function. Machine perfusion techniques (normo-thermic machine perfusion with oxygenation or hypothermic machine perfusion with or without oxygenation) are an important strategy to improve transplant outcomes, and these systems aspire to improve the condition of marginal organs and optimise the quality of standard criteria donor organs. Machine perfusion may be continued throughout transport if desired, and specialist equipment is available for this purpose. It may also be used as a method of reconditioning organs prior to transplant which is especially pertinent for organs which have been kept in static cold storage. Different approaches have been used for different organs, and reconditioning using machine perfusion also permits administration of medications which may aid the process. In order to ensure equity of access, it is crucial that these strategies are supported both logistically and financially by the NTO.

Organ sharing between units and regions should be dictated by nationally-approved rules and criteria and coordinated by regional NTO offices under the central supervision of this organization.

#### Live Donation (LD)

To achieve national self-sufficiency with respect to organ donation and transplantation, DD and LD should be seen as complementary processes (10, 18, 57). A pre-emptive transplant from an LD should be the treatment of choice for patients with renal failure. International evidence has documented excellent short and long-term outcomes after LD with respect to both clinical results and patient quality of life (63, 65, 79).

LD may be from a related (genetically or emotionally) or unrelated donor and may also include exchange programs and altruistic or anonymous donations. Most living donors are related in some way to the recipient; altruistic donation is a largely underutilized mode in most countries (80). A focus on altruistic donation may serve to expand the donor pool and may represent a source of organs for difficult or rare matches, especially when incorporated into a wider organ exchange scheme. In the UK in 2019, more than 100 individuals altruistically donated a kidney; many of these donations were incorporated into the UK Living Kidney Sharing Scheme (81).

The safety and protection of LDs are of paramount importance. The WHO Guiding Principles on Human Cell, Tissue, and Organ Transplantation (15), the Declaration of Istanbul (17), and the Council of Europe Convention against Trafficking in Human Organs (82) all provide standards and guidance on this matter.

Several internationally-recognized documents provide detailed guidance on the assessment of LDs (7, 10, 12) and the provision of follow-up for these individuals (83–85). LD should be cost-neutral and should not present a financial burden to the donor. There are currently several international examples of useful donor reimbursement schemes from countries with high LD rates including the UK (86), Israel (87), and the Netherlands (88, 89). Example 7 provides additional information on the UK LD reimbursement scheme.

Example 7 Making LD cost-neutral in the UK: the NHSBT living donor reimbursement scheme

The NHSBT living donor reimbursement scheme was carefully designed to account for any direct or indirect financial burden that may be incurred as part of the process of donating an organ. The scheme covers:

• Travel expenses (including any tolls or charges)

• Loss of earnings from employment (including over-time) or self-employment

• Loss of any state benefits

• Accommodations

• Childcare or other dependent care.

• Any medical expenses incurred that were not otherwise covered.

• Costs of temporary staffing for businesses

• Donation-related prescription costs (86)

Dialysis assessments should always include the possibility of identifying an LD. Referring specialists (most notably, nephrologists) play a critical role in providing referrals and assessments for LD (90, 91).

Unfortunately, up to 40% of recipients find they are incompatible with their intended donors (92); thus, the implementation of a kidney exchange scheme may significantly boost the rate of LDs. Spain, the Netherlands, and the UK all provide examples of well-developed kidney exchange schemes (92).

#### Transplantation

The multidisciplinary nature of transplantation necessitates careful workforce planning to ensure the availability of sufficient and appropriately trained staff from all disciplines that can provide consistent coverage at all times. Multidisciplinary teams must be employed to perform pre-transplant assessments and re-assessments of patients remaining on the waiting list. This can be facilitated *via* the use of sophisticated IT systems (as described above). The NTO should maintain overall responsibility for the coordination of this process, particularly with respect to organ matching and allocation, and must have appropriately-trained staff available at all times to provide support and advice.

Due to the need to act swiftly to preserve organ viability, crucial support must be provided to maintain close collaboration and communication between the many individuals involved in organ transplantation. All aspects of transplantation, including waiting list assessments and decisions, organ matching, allocation, offering, perioperative management, transplant surgery, and post-transplant hospitalization must be addressed using nationally-approved protocols and guidance. Different countries have taken varying approaches to ensure the coordination of the many processes involved in achieving successful transplantation. Example 8 describes the well-established system and the role of Transplant Recipient Coordinators in the UK.

Example 8 The role of the Transplant Recipient Coordinator: the UK.

The role of the Transplant Recipient Coordinator is fully embedded in the UK program. There are currently more than 250 Transplant Recipient Coordinators assigned to the 27 transplant units in the UK. These professionals are typically specialist nurses who provide support and advice to transplant recipients throughout the entire process, from referral through long-term follow-up. An appointed lead nurse is assigned to represent and support the Transplant Recipient Coordinators in all aspects of their work. This individual is expected to maintain close relationships and open channels of communication with key stakeholders, including the NHSBT, solid organ advisory groups, transplant units, and patient groups (93).

#### Post-Transplant Follow-Up

Long-term follow-up must be provided by multidisciplinary organ-specific teams. Several internationally recognized documents provide guidance on ideal follow-up arrangements for post-transplant patients. These documents provide specific recommendations regarding renal (8, 9, 94, 95), liver (96–98), and heart/heart-lung (99, 100) transplantation procedures. Table 4 details some of the key principles involved in post-transplant care.

**TABLE 4:** Key principles for follow-up post-transplant.

• National guidelines based on international best practices
• Regular reviews and assessments
• Shared-care arrangements for those living in remote locations that can be facilitated by telemedicine technology
• Immunosuppressive protocols and optimization of immunosuppressive therapy
• Efforts to prevent recurrence of disease (e.g., management of hypertension, diabetes, inflammatory disorders)
• Management and minimization of post-transplant-related complications
• Optimization of psychosocial outcomes
• Recording and disseminating nationally reviewed and approved outcome data

### Research and Training

#### Research and Development

All staff members involved in organ donation and transplantation should be encouraged to participate in research activities. It may be helpful to establish a research division within the NTO similar to the program included in the NHSBT (101) and/or develop collaborations with international research programs (e.g., Eurotransplant) (102). Research funding may be obtained *via* granting mechanisms, partnerships with private organizations, and/or participation in international consortia (e.g., the European Society for Organ Transplantation [ESOT]) (103).

A proposal on the standards of quality and safety of substances of human origin (SoHO) was adopted by the European Commission in July 2022. This proposal repeals the current legislation and aims to update and improve the regulations for the quality and safety of SoHO. This new legislation also intends to support research, innovation, cross-border learning and rapid adoption of evidence-based developments while offering the highest degree of protection and safety to donors and recipients (104).

#### Teaching, Training, and Professional Development

All staff members must have access to high-quality training and regular updates. This includes staff members who might engage with potential donors as well as physicians caring for patients with organ failure. Training in communication skills is of the utmost importance; simulation training is effective for this purpose (105, 106). Employment plans should incorporate adequate time for training activities. Individually-tailored continuing professional development portfolios with a personal development plan and annual (or more frequent) appraisals should be basic standard requirements. Clear arrangements for supervision should be instituted for all junior staff. The ODEQUS suggests that an annual hospital-wide seminar on organ donation should be organized (5) and that organ donation and transplantation should be included in both medical and nursing school curricula (5). Example 9 outlines some of the internationally-respected training opportunities available to healthcare professionals.

Example 9 Internationally-respected training opportunities.

Internationally-respected training opportunities are available for all professionals involved in organ donation and transplantation. The European Union of Medical Specialists (UEMS) provides comprehensive training and accreditation programs. Similarly, the European Society for Organ Transplantation (ESOT) offers extensive educational portfolios and support, and the Spanish Transplant Procurement Management-Donation & Transplantation Institute (TPM-DTI) foundation provides many highly-regarded training and research opportunities (107).

#### Scientific Societies and Professional Organizations

These bodies are invaluable sources of expert advice, opinion, and support and they provide opportunities for professionals to meet and exchange experiences and ideas. They also support and fund research and help to disseminate the results and relevant outcomes *via* publications and educational events. They set professional standards and codes of conduct and provide accredited training programs. Their advice should be sought in the development of guidelines and protocols and matters relating to changes in legislation, regulation, and complex ethical issues.

## Discussion

The organ donation and transplantation framework described in this paper is a helpful tool that can be adapted by countries seeking to plan a new program or evaluate one that is already in existence. While some elements may seem to be more important than others, they are all interconnected and interdependent. Improvements to a program will only be realized in response to system-wide change and close collaboration between all parties. Moreover, it may not be possible to address specific areas within the framework (for example, prevention) in the absence of wider health-system reform. Gaps between policy plans and policy implementation are frequently observed. Thus, while the application of this framework may help to guide policy development, its overall implementation must be viewed as a long-term and ongoing project.

One strength of this framework is that it was developed with input from a panel of international experts in organ donation and transplantation; these individuals participated actively in the process of validating the components of the framework and provided constructive and insightful feedback. The research team also had the opportunity to apply the framework to six nation-specific case studies performed in parallel with this study that provided further validation of these results (18–23).

However, there are several limitations to consider. First, although we attempted to create domains that could be generalized for a wide variety of circumstances, certain components will certainly need some adaptation when applied in different settings. For example, we recognize that this framework was devised and constructed based on best practice evidence from high-income countries. We understand that significant modifications would be required to adapt this framework for use in low- or middle-income countries. Second, the framework attempts to take a wide view of the theme of organ donation and transplantation by embracing many elements which may influence this process. However, as this topic involves many different disciplines, there may be certain aspects that we have neglected to address.

Despite these limitations, we expect that this framework can be adapted to different settings specifically in high-income countries. Some components may need to be modified to suit individual contexts and needs by taking into account cultural and societal norms as well as political and economic circumstances. This will be especially important when considering ethically challenging areas, for example, consent legislation, diagnosis of death, DCD, and the withdrawal of life-saving treatment. The involvement of specific stakeholders and the nature of educational or publicity campaigns will also be highly dependent on national circumstances and sensibilities. We also recognize that no program exists in isolation and that all countries will be building on and improving some type of existing structure. This concept relates not only to the donation and transplantation program itself but also to the wider capacities of a given healthcare system. For example, the extent to which different jurisdictions can build effective preventative strategies will depend largely on the developmental state of existing public health and primary care systems. Likewise, the speed at which appropriate digital systems can be organized will depend directly on the stage of progress of existing healthcare IT.

Finally, the framework offers countries the opportunity to learn from one another. The use of this framework will not only highlight areas in need of improvement, but also examples of good and innovative practices. Valuable lessons may also be exchanged, including strategies that have not led to success and/or those resulting in unintended negative consequences.

## Data Availability

The raw data supporting the conclusion of this article will be made available by the authors, without undue reservation.

## Appendix

### Members of the expert panel

Dr. Mirela Bušić, Ministry of Social Affairs and Health, Croatia.

Professor Daniel Casanova, University of Cantabria, Spain.

Dr. Ana Franca, Instituto Português do Sangue e da Transplantação, Portugal.

Dr. Aliki G. Iniotaki, General Hospital of Athens G. Gennimatas, Greece.

Professor Anastasia Kotanidou, National and Kapodistrian University of Athens, Greece.

Dr Jorge Paulino Pereira, University of Lisbon, Portugal.

Professor Jacopo Romagnoli, Catholic University of Rome, Italy.
